# Monitoring Oral Hygiene in Inpatient Psychiatric Units: A Clinical Audit

**DOI:** 10.1192/bjo.2026.11662

**Published:** 2026-06-30

**Authors:** Zaim Mohdesham, Kopal Tandon, Ahmed Rozza, Jasmeet Sidhu, Noor Us-Saba

**Affiliations:** Derbyshire Healthcare NHS Foundation Trust, Derby, United Kingdom

## Abstract

**Aims:**

Individuals with severe mental illness experience a disproportionate burden of oral disease. Contributory factors include medication-induced xerostomia, cognitive deficits, and impaired motivation for self-care. Studies found that they are significantly less likely to engage in preventative dental behaviours or utilize services. Inpatient admission provides an opportunity to engage our service users in promoting their oral hygiene. Hence, an audit can provide valuable insights into current practices and identify areas for improvement and highlight gaps in service provision.

**Methods:**

The study was conducted between April to May 2025 across inpatient mental health units within Derbyshire Healthcare NHS Foundation Trust. Electronic patient records were reviewed to assess completion of the ‘Eating, Drinking and Oral Health’ questionnaire and the standardized oral health risk assessment during their admission. The audit also examined the prevalence of documented dental issues and access to dental services. A structured data collection tool was developed to capture sex, gender, ward type, documentation of oral health assessments, completion of dental screening questionnaires and risk assessments, identified oral health issues, and access to dental care during their admission. Trust inpatient policy requires all inpatients to receive a comprehensive physical health assessment including oral health examination on admission, and dental hygiene included in ongoing nursing care reviews.

**Results:**

A total of 114 patients were included in this study, from acute working age (n=40), older adult (n=34), low-secure forensic (n=20), and rehabilitation ward (n=19), with a mean age of 53.6 years. 72/114 were male. Although the “Eating, Drinking and Oral Health” questionnaire was partially completed for 61.4% (n=70/114), full compliance was not achieved for any patient. Formal oral health examinations were recorded for only 10.5% (n=12/114), with the lowest rates observed in the forensic unit (0%). Notably, no patients had a completed oral health risk assessment. Of the 30 patients who presented with acute dental issues during their admission, 18 successfully accessed professional dental care, though access rates varied significantly between ward types with the service users in the forensic ward among the highest.

**Conclusion:**

The audit demonstrates that current monitoring and documentation practices of oral hygiene in our local trust requires improvement. Immediate interventions should focus on increasing awareness, optimizing the accessibility of relevant assessment tools, and implementing clinical prompts to improve documentation quality. Long-term strategies must prioritize the integration of oral health into multidisciplinary care planning and the establishment of formal referral pathways with community dental providers to ensure holistic patient management.

